# Metastasis of osteosarcoma to stomach made clinically evident by hematemesis: a case report

**DOI:** 10.1186/1477-7819-11-48

**Published:** 2013-02-26

**Authors:** Hiroshi Urakawa, Satoshi Tsukushi, Issei Tsurudome, Akihiro Hirata, Eisuke Arai, Eiji Kozawa, Naohisa Futamura, Ryoji Miyahara, Naoki Ishiguro, Yoshihiro Nishida

**Affiliations:** 1 Department of Orthopedic Surgery, Nagoya University Graduate School and School of Medicine, 65 Tsurumai, Showa-ku, 466-8550, Nagoya, Aichi, Japan; 2 Department of Gastroenterology and Hepatology, Nagoya University Graduate School and School of Medicine, 65 Tsurumai, Showa-ku, 466-8550, Nagoya, Aichi, Japan; 3 Division of Surgical Oncology, Department of Surgery, Nagoya University Graduate School and School of Medicine, 65 Tsurumai, Showa-ku, 466-8550, Nagoya, Aichi, Japan

**Keywords:** Osteosarcoma, Metastasis, Stomach, Hematemesis

## Abstract

**Background:**

Gastric metastasis from osteosarcoma is very rare and its clinical features are not well recognized.

**Case presentation:**

A 73-year-old man was diagnosed with osteosarcoma and treated with four cycles of preoperative chemotherapy with ifosfamide and doxorubicin followed by wide resection. Two cycles of postoperative chemotherapy with ifosfamide and doxorubicin and ten cycles of chemotherapy with carboplatin and etoposide were administered. Eleven months after the surgery, he vomited fresh blood. Unusual progression of anemia was observed with the hematemesis. A biopsy was performed by gastrointestinal endoscopy, and the stomach tumor was diagnosed as metastasis of osteosarcoma.

**Conclusions:**

Even though gastric metastasis from osteosarcoma is very rare, all three previous reports and our case showed the presence of ulcer on the surface of the gastric lesion. We should consider the possibility of gastric metastasis in patients with osteosarcoma in whom progression of anemia or gastric hemorrhage is observed.

## Background

Osteosarcoma is the most common primary bone tumor in children and adolescents [1,2]. But, recently the incidence of primary osteosarcomas has also been increasing in older persons [3]. Osteosarcoma is a highly metastatic tumor, whose most common metastatic site is the lung [4]. Gastric metastasis of osteosarcoma is very rare [5-7], and its clinical features are not well recognized. We detected gastric metastasis in a 73-year-old man, and report here for the first time gastric metastasis from a trunk osteosarcoma in an older person.

## Case presentation

**Figure 1:**
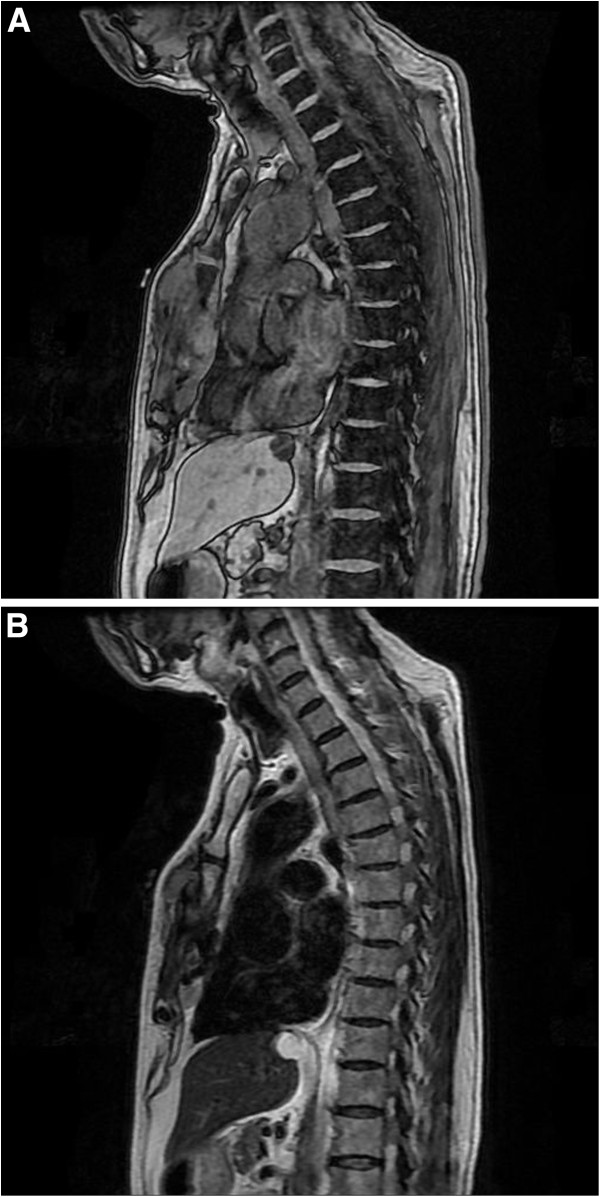
Magnetic resonance T1-weighted image (A) and T2-weighted image (B) of primary site of sternum.

**Figure 2:**
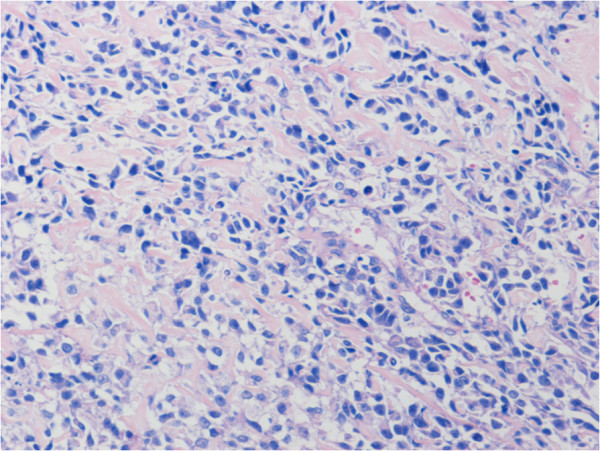
**Microphotograph of biopsy specimen in primary site of sternum (H & E. ×200). **Pleomorphic spindle-shaped atypical cells were observed with osteoid.

**Figure 3:**
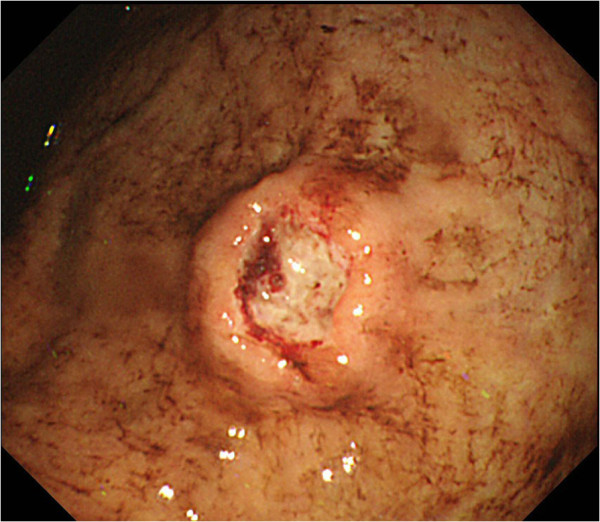
Upper gastrointestinal endoscopic image revealed a tumor with bleeding ulceration on the gastric body.

**Figure 4:**
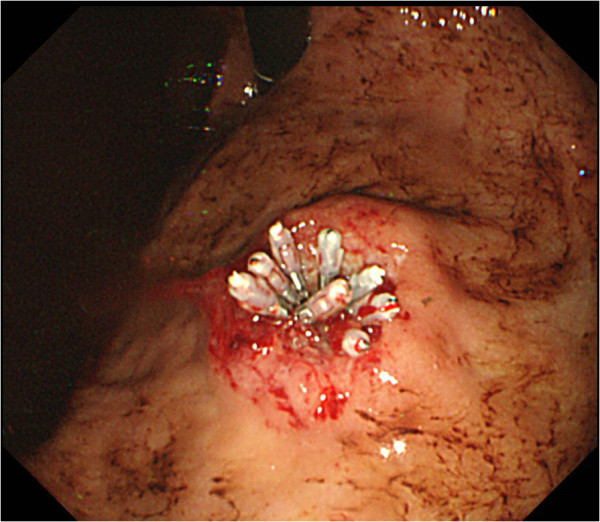
An endoscopic image of tumor ulceration after staunching procedure by clippings and injections of ethanol and hypertonic saline-epinephrine.

**Figure 5:**
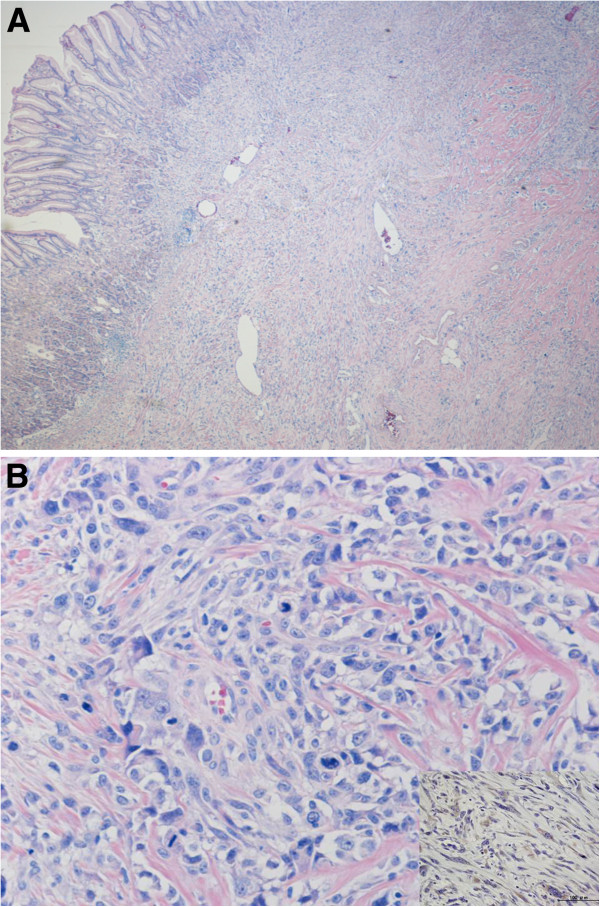
**Microphotograph of resected specimen in gastric metastasis (H & E. ×20 (A), ×200 (B), ×400 (B inset)). **Tumor was observed under the gastric mucosa (**a**), and pleomorphic spindle-shaped atypical cells with osteoid were observed (**b**). Most osteosarcoma cells expressed VEGF in the gastric metastasis (b inset). VEGF, vascular endothelial growth factor.

A 73-year-old man consulted a general hospital with a chief complaint of a hard mass in his precordia. He consulted our university hospital one month later. He was diagnosed with localized osteosarcoma of the sternum by clinical imaging (Figure 1A, B) and needle biopsy. Histological examination of the biopsy specimens revealed pleomorphic spindle-shaped atypical cells with osteoid, and he was diagnosed with osteosarcoma (Figure 2). He was treated with 4 cycles of preoperative chemotherapy with ifosfamide 2 g/m^2^ on days 1 to 5 and doxorubicin 20 mg/m^2^ on days1 to 3 every 3 weeks followed by wide resection which involved the sternum, bilateral costal cartilages, and pleura, chest wall reconstruction with mesh, and plastic reconstruction with a latissimus dorsi muscle free flap. Histological examination of the resected specimens revealed a necrosis rate of 95% and a microscopically negative surgical margin. Pleural effusion was observed after surgery, and fine needle aspiration cytology revealed tumor cells in the pleural effusion. Two cycles of postoperative chemotherapy with ifosfamide and doxorubicin and 10 cycles of chemotherapy with carboplatin 300 mg/m^2^ on day 1 and etoposide 100 mg/m^2^ on day 1 were administered every 4 weeks. No obvious lung or pleural metastasis was observed on chest computed tomography (CT) after these treatments. Eleven months after the surgery, blood tests showed a mild decrease of hemoglobin from 8.6 to 7.8 g/dl in one week, and he vomited fresh blood at the toilet of our hospital on the day of the blood test. Gastrointestinal endoscopic examination was performed promptly and showed a tumor on the gastric body that was like a type IIa + IIc gastric cancer (Figure 3). Fresh bleeding was observed at an ulcer on the tumor surface, and staunching was performed with clippings and injections of ethanol and hypertonic saline-epinephrine (HSE) (Figure 4). Two units of red-cell transfusions derived from 400 ml of whole blood were administered. Six days later, biopsy was performed by gastrointestinal endoscopy, and histological examination of the biopsy specimens revealed pleomorphic atypical cells with osteoid, and the diagnosis of the stomach tumor was metastasis from osteosarcoma. Partial gastrectomy with a wide margin was performed by laparoscopy to prevent re-bleeding, and the histological diagnosis was the same as that of the biopsy (Figure 5A, B). Most osteosarcoma cells expressed vascular endothelial growth factor (VEGF) in the gastric metastasis (Figure 5B inset). One month after the partial gastrectomy, a single lung metastasis was observed on CT, for which stereotactic radiation therapy was performed as a palliative therapy. The patient remains stable as of eight months after the radiation to the lung.

## Discussion

Bacci *et al*. reported recurrence in 313 of 789 osteosarcoma patients (39.7%) after neoadjuvant chemotherapy, with the most common primary recurrence site being lung (77.6%), followed by bone (8.3%), local (6.4%), local and bone (2.6%), lung and bone (1.6%), kidney (0.9%), brain (0.9%), and heart (0.9%) [4]. Palma *et al*. reported 64 cases with gastric metastases from primary sites including breast (n = 21, 32.8%), lung (n = 16, 25.0%), malignant melanoma (n = 14, 21.9%), head and neck (n = 4, 6.2), and uterus (n = 4, 6.2%), but none from osteosarcoma [8]. Gastric metastasis of osteosarcoma is very rare, with only three such previous reports available [5-7]. Our case was different from the others with respect to age and primary site because the present case was older with a trunk primary (Table 1), and the difference may reflect the increasing number of older trunk osteosarcoma patients [3]. Past reports have shown that osteosarcoma gastric metastasis occurred with simultaneous or previous lung metastases [5-7]. In our case, lung metastasis was observed one month after partial gastrectomy, and we considered that the gastric metastasis simultaneously occurred with the lung metastasis.

**Table 1:** Previous reports of osteosarcoma gastric metastasis

**Authors**	**Age**^**a)**^	**Sex**	**Primary site**	**Duration**^**b)**^**, months**	**Size**^**c)**^**, cm**	**Ulcer on surface**	**Symptoms**
Overberg-Schmidt *et al*.	11	female	femor	14	4	Yes	hematemesis, anemia
Strong *et al*.	17	male	femor	30	3	Yes	tarry stools, vomiting, anemia
Horiuchi *et al*.	15	male	humerus	30	-	Yes	abdominal pain
Our case	73	male	sternum	11	2.5	Yes	hematemesis, asymptomatic anemia

^a^Age at diagnosis of primary tumor; ^b^duration from operation of primary tumor to diagnosis of gastric metastasis; ^c^size of gastric metastasis.

Grimer *et al*. reported that patients over the age of 60 years with high grade conventional osteosarcoma received less chemotherapy and fared worse than patients under the age of 60 years in a retrospective review of patients over the age of 40 years with osteosarcoma [9]. Chemotherapy regimens based on effect, risk, and compliance would be important to limit the incidence of side effects in older osteosarcoma patients, but no chemotherapy protocol has been established specifically for this group. Bacci *et al*. reported that neoadjuvant chemotherapy with ifosfamide, doxorubicin, and cisplatin for patients 41- to 60-years old yielded a similar clinical outcome to that of patients less than 40 years of age who were treated with conventional chemotherapy [10]. Since the completion rate of regimens which included cisplatin and/or methotrexate has been reported to be low in middle-aged and older patients [11,12], we administered preoperative and postoperative chemotherapy with ifosfamide and doxorubicin. We also used carboplatin and etoposide as a palliative chemotherapy after postoperative recurrence because ambulatory treatment is possible for these drugs.

Menuck *et al*. reported 17 gastric metastases in autopsies of 1,010 patients with cancers; 10 of these 17 did not have clinical manifestations [13]. Interestingly, all previous reports showed that gastric metastases of osteosarcoma had ulceration on the surface of the gastric metastasis, as in our case, while three of four cases had anemia and all had clinical manifestations [5-7]. In these previous reports of osteosarcoma gastric metastasis, gastric tumor excision was performed in two of three cases with a wide or radical margin [6,7]. In our case, partial gastrectomy was performed by laparoscopy to prevent re-bleeding from the ulcerated portion of the gastric metastasis. Previous reports and our case indicate that gastrectomy was performed of necessity in patients who had symptoms of ulcer hemorrhage.

VEGF expression in osteosarcoma was reported to be associated with a high potential for pulmonary metastasis [14]. In our case, the gastric osteosarcoma metastasis expressed abundant VEGF promoting the formation of new blood vessels out of a preexisting vascular network. Immature new vessels may be associated with easy bleeding from the ulcer on the osteosarcoma gastric metastasis. Ulceration is not common in skin metastases of osteosarcoma [15], but is distinctive for gastric metastases of osteosarcoma. Local factors at the metastatic site may also be implicated in the occurrence of ulceration.

## Conclusions

Summing up, we observed gastric metastasis from a trunk osteosarcoma in an older patient. Even though osteosarcoma gastric metastasis is infrequent, progression of anemia or gastric hemorrhage in the follow up of osteosarcoma should raise suspicion of a gastric metastasis.

## Consent

Written informed consent was obtained from the patient for publication of this case report and any accompanying images. A copy of the written consent is available for review by the editor-in-chief of this journal.

## Abbreviations

CT: Computed tomography;H & E: Hematoxylin and eosin;HSE: Hypertonic saline-epinephrine;VEGF: Vascular endothelial growth factor.

## Competing interests

The authors declare that they have no competing interests.

## Authors’ contributions

HU designed and drafted the manuscript; YN developed the concept and treatment of the osteosarcoma; NI revised the manuscript and supervised; ST, EA, EK, and NF treated the osteosarcoma; IT and RM gave the endoscopic diagnosis; AH performed the surgical treatment of the gastric metastasis. All authors read and approved the final manuscript.
